# Exploring C_4_–CAM plasticity within the *Portulaca oleracea* complex

**DOI:** 10.1038/s41598-020-71012-y

**Published:** 2020-08-28

**Authors:** Renata Callegari Ferrari, Bruna Coelho Cruz, Vinícius Daguano Gastaldi, Thalyson Storl, Elisa Callegari Ferrari, Susanna F. Boxall, James Hartwell, Luciano Freschi

**Affiliations:** 1 Departamento de Botânica, Instituto de Biociências, Universidade de São Paulo, São Paulo, Brasil; 2 Department of Functional and Comparative Genomics, Institute of Integrative Biology, University of Liverpool, Liverpool, UK

**Keywords:** Plant physiology, Abiotic, Drought, C4 photosynthesis

## Abstract

*Portulaca oleracea* is a C_4_ herb capable of performing CAM under drought stress. It is distributed worldwide and is either considered a polymorphic species or a complex of subspecies, due to its numerous morphological variations. We evaluated CAM plasticity within *P. oleracea* genotypes since the complexity surrounding this species may be reflected in intraspecific variations in photosynthetic behavior. Eleven subspecies of *P. oleracea* from distant geographical locations and one cultivar were morphologically and physiologically characterized. C_4_ and CAM photosynthesis were monitored in plants exposed to well-watered, droughted and rewatered treatments, and data obtained were compared among individual genotypes. All subspecies expressed CAM in a fully-reversible manner. Transcript abundance of C_4_–CAM signature genes was shown to be a useful indicator of the C_4_–CAM–C_4_ switches in all genotypes. C_4_-related genes were down-regulated and subsequently fully expressed upon drought and rewatering, respectively. CAM-marker genes followed the opposite pattern. A gradient of morphological traits and drought-induced nighttime malate accumulation was observed across genotypes. Therefore, different combinations of CAM expression levels, plant sizes and shapes are available within the *P. oleracea* complex, which can be a valuable tool in the context of C_4_/CAM photosynthesis research.

## Introduction

C_4_ photosynthesis and the crassulacean acid metabolism (CAM) are carbon (C) concentrating mechanisms (CCMs), similar in their biochemical pathways, as both use phospho*enol*pyruvate carboxylase (PPC) to perform the primary fixation of CO_2_ into 4-C acids^1,2^. These acids are subsequently decarboxylated, regenerating CO_2_ in the vicinity of ribulose-1,5-biphosphate carboxylase/oxygenase (Rubisco) to minimize O_2_ binding. The oxygenase activity leads to 3-phosphoglycerate (3PGA) and 2-phosphoglycolate (2PG) formation, the latter molecule being toxic, and requiring processing and elimination via photorespiration^3^. Despite the shared similarities between C_4_ and CAM, each CCM is usually associated with a specific set of anatomical characteristics and regulatory mechanisms, rendering uncommon the co-occurrence of both syndromes within a single plant^4^.

Overall, C_4_ acts as a spatial specialization involving the transfer of CO_2_ acceptor molecules between a mesophyll and a bundle sheath cell (MC and BSC, respectively)^5^. In contrast, CAM works as a temporal specialization, where acid formation and mobilization occur in a single MC but at different times of the day^6^. Also, CAM has been shown to vary in the degree of diel acid fluctuation and gas exchange patterns, with the contribution of the nocturnal CO_2_ primary fixation to total C assimilation varying across species^7^. These features characterize different types of CAM, ranging from strong CAM, where virtually all CO_2_ assimilation derives from CAM activity^8^ (Nobel 1988), to weak CAM (e.g., CAM cycling), in which atmospheric CO_2_ uptake takes place exclusively during the day, and the refixation of respiratory CO_2_ leads to a small nighttime accumulation of organic acids^9^.

At the weak-CAM end of the spectrum, *Portulaca* species are C_3_–C_4_ intermediates and C_4_ plants capable of performing CAM under drought. Facultative CAM was shown to occur in species belonging to the six phylogenetic clades of *Portulaca*, some of which performing CAM-cycling when water is deprived^10–16^. In addition to this complex scenario, different decarboxylating systems occur in *Portulaca*, with NADP-malic enzyme (ME) and NAD-ME representatives such as *P. grandiflora* and *P. oleracea*, respectively^17,18^.

*Portulaca oleracea* is a promising candidate for a C_4_/CAM model species due to its fast growth, efficient seed production, and accumulating literature on CCM-related biochemical and gene expression data^19–26^. The uncommon C_4_-to-CAM transition in leaves of *P. oleracea* is associated with the transcriptional induction of specific genes. They include a CAM-specific PPC isoform (*PPC-1E1c*), an aluminum-activated malate transporter (*ALMT-12E.1*) and a dicarboxylate carrier (*DIC-1.1*)^23,27^, and their relative transcript abundances have been suggested as a valuable tool to assess CAM induction in this species^27^. ALMT proteins have a role in nocturnal malate uptake into the vacuole^28,29^, while DIC transporters mediate C-skeleton transport across mitochondria^30^. Besides, several C_4_-related transcripts were identified to be exclusively expressed in leaves under well-watered conditions in *P. oleracea*, including a C_4_-related PPC isoform (*PPC-1E1a’*)^23^, a NAD-ME (*NADME-2E.1*) and an aspartate aminotransferase (*ASPAT-1E1*)^27^.

Commonly known as purslane, *P. oleracea* can germinate over a wide temperature range (10–40 °C)^31^, thriving in various light intensities, photoperiods, soil types and moisture conditions^32–34^, even being considered a noxious weed for agriculture^35^. It also presents a cosmopolitan distribution, occurring in most tropical and subtropical regions^36^. Variations in chromosome number^37^, vegetative and reproductive morphology^38–42^ are already described between accessions of this species. Because of such high phenotypical plasticity, *P. oleracea* is sometimes referred to as a polymorphic species^33,37^, or even subdivided into different subspecies^43,44^ or microspecies^45–47^ that form a taxonomic aggregate or complex. The most up-to-date phylogeny detailing *Portulaca* infra-familiar relationships still refers to the traditional *P. oleracea* subspecies system to highlight that this clade is paraphyletic and clusters with other species, e.g. *P. molokiniensis*^44,48^*.*

However, comparative information on the physiological performance, particularly in the context of CAM plasticity and CCM-related transcriptional reprogramming, is missing for the *P. oleracea* complex. Understanding CAM plasticity across *P. oleracea* genotypes may be a valuable source of information for future biotechnological applications seeking to explore C_4_/CAM compatibility using this species as a model^26,49^. Therefore, we hypothesized that there might be different degrees of CAM expression among members of the *P. oleracea* complex, particularly when comparing genotypes with distinctive morphological traits (e.g., leaf succulence and size) and originally from contrasting environmental conditions.

To this end, we assembled a collection of twelve *P. oleracea* genotypes, composed of eleven subspecies from different geographic regions and one cultivar, to compare physiological variation in CAM response. Subspecies were characterized based on climatic conditions of their place of origin or morphological traits using a clustering approach by principal component and hierarchical clustering analyses (PCA and HCA, respectively). Nighttime malate accumulation (Δ_malate_) and transcript abundance of CAM- and C_4_-signature genes revealed that weakly expressed, facultative CAM is a shared trait among all genotypes analyzed. We also validated the previously published recommendation of specific C_4_ and CAM genes^27^ by monitoring their relative transcript abundance and detecting similar expression patterns across various genotypes. Finally, our findings indicate that different combinations of drought-induced CAM expression intensities, plant sizes and shapes are available within the *P. oleracea* complex, an array that offers various possibilities for future C_4_/CAM photosynthesis research.

## Results

### Characterizing *P. oleracea* genotypes using clustering approaches

Previously identified *P. oleracea* subspecies and one cultivar genotype (Table 1) were grown side-by-side under greenhouse conditions for three generations before the start of the experiments. Seed attributes were analyzed under scanning electron microscopy (SEM) to confirm subspecies identification and the purity of lots (Supplementary Figure S1). A commercial cultivar was included as a reference genotype for physiological analyses since it was previously used in physio-transcriptomic studies^27^. Additional Brazilian wild accessions were identified via SEM analysis either as subsp. *granulatostellulata* or subsp. *nitida* (Supplementary Figure S1), and were not included in the physiological experiments, as these subspecies were already represented in our collection.

**Table 1:** Identification and geographical origin of *Portulaca oleracea* genotypes.

Genotype identification	Taxonomic identification^a^	Geographical origin	Refs.
*trituberculata*	*Portulaca trituberculata *Danin, Domina & Raimondo	Greece, 38° 13′37′′ N, 25° 59′57′′ E	[1]
*sicula*	*Portulaca sicula *Danin, Domina & Raimondo	Italy, 43° 27′53′′ N, 11° 52′39′′ E	[1]
*oleracea*	*Portulaca oleracea *subsp. *oleracea*/*Portulaca* *trituberculata* [1]	Chile, 36° 49′ S, 73°03′ W	[1]
*rausii*	*Portulaca rausii *Danin	Greece, 36° 50′56′′ N, 27° 04′31′′ E	[1]
*zaffranii*	*Portulaca zaffranii* Danin	South Africa, 23° 04′04,1′′ E, 34° 02′36,5′′ S	[1]
*nitida*	*Portulaca nitida* (Danin & H.G.Baker) Ricceri & Arrigoni	Israel, 31° 47′11′′ N, 34° 42′33′′ E	[2]
*edulis*	*Portulaca edulis* Danin et Bagella [2]	Greece, 35° 05′08′′ N, 33° 16′46′′ E	[1]
*papillatostellulata*	*Portulaca papillatostellulata* (Danin & H.G.Baker) Danin	Austria, 48° 17′43′′ N, 16° 52′18′′ E	[1]
*sativa*	*Portulaca oleracea* subsp. *sativ*a (Haw.) Čelak	Austria, 48° 11′33′′ N, 16° 23′04′′ E	[1]
*tuberculata*	*Portulaca tuberculata* León	Peru, 12° 35′39′′ S, 69° 11′38′′ W	[1]
*granulatostellulata*	*Portulaca granulatostellulata* (Poelln.) Ricceri & Arrigoni	Israel, 32° 23′60′′ N, 34° 52′58′′ E	[2]
cultivar	commercial cultivar	Agristar^b^	[3]

^a^Taxonomic identification according to the registry at The Plant List (2013), except for *P. edulis* since it is not listed. [1] Walter et al., 2015. [2] Danin et al., 2012. [3] Ferrari et al. 2020.

^b^Seeds bought from Agristar do Brasil Ltda.—São Paulo, Brazil.

The climatic conditions of the place of origin and morphological attributes were compared for the eleven subspecies, and the cultivar was included in the morphological analysis only. Data obtained was analyzed via principal component analysis (PCA) for cluster identification, and then further evaluated using hierarchical clustering analysis (HCA).

**Figure 1:**
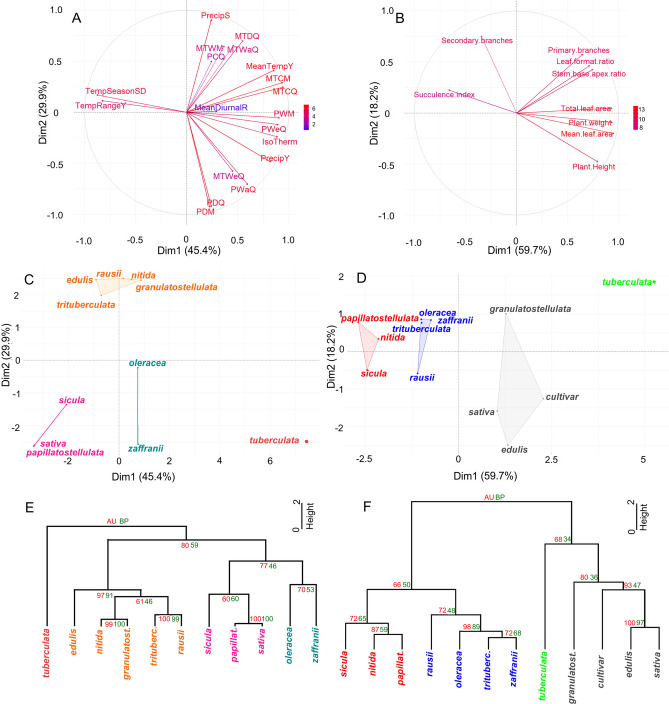
Characterization of the *Portulaca oleracea* complex based on climatic and morphometric variables via principal component analysis (PCA) and hierarchical clustering analysis (HCA). (**A,B**) Variable contribution to each PC. The first two PCs explain 75% and 77% of the variance in A and B, respectively. The color scale indicates the relative variable contribution to each PC. (**C,D**) Groups of subspecies formed—the first two PCs harbor the most significant correlations to the variables. (**E,F**) HCA groups subspecies into clusters and values of approximately unbiased (AU) and bootstrap (BP) are presented in red and green, respectively (see Materials and methods section for details). Data for these analyses are presented in Tables S1 and S2. In (**A,C,E**), the following climatic variables were analyzed: latitude, longitutude, annual mean temperature (MeanTempY), mean diurnal range (MeanDiurnalR), isothermality (IsoTherm), temperature seasonality standard deviation (TempSeasonSD), max. temperature of warmest month (MTWM), min. temperature of coldest month (MTCM), temperature annual range (TempRangeY), mean temperature of wettest quarter (MTWeQ), mean temperature of driest quarter (MTDQ), mean temperature of warmest quarter (MTWaQ), mean temperature of coldest quarter (MTCQ), annual precipitation (PrecipY), precipitation of wettest month (PWM), precipitation of driest month (PDM), precipitation seasonality (PrecipS), precipitation of wettest quarter (PWeQ), precipitation of driest quarter (PDQ), precipitation of warmest quarter (PWaQ), precipitation of coldest quarter (PCQ).

First, 19 climatic variables were retrieved from the World Clim database^50^ using the geographic coordinates from the sampling place of each subspecies (Supplementary Table S1, Fig. 1A,C,E). Variables presented >|0.64| correlations to the first 4 PCs, but we focused on PC 1 and 2 since 16 out of the 19 variables were significantly correlated to these PCs. One genotype, *tuberculata*, was isolated from the other subspecies in PC1 by scoring high in winter temperature parameters. On the other hand, precipitation variables reflecting on seasonality contributed to separating the remaining subspecies along PC2. The following groups were formed: 1—consisting of *tuberculata* alone, 2—formed by *edulis, trituberculata, rausii, nitida* and *granulatostellulata*; 3—formed by *sicula, sativa* and *papillatostellulata*; 4—including *oleracea* and *zaffranii* (Fig. 1C,E).

Second, nine morphometric parameters were measured in the 12 genotypes, using at least 25 well-watered, two-month-old individuals that were grown side-by-side under controlled conditions (Supplementary Table S2, Fig. 1B,D,F). These data were subsequently analyzed by PCA and HCA. The first three principal components explained a total of 90.16% of the variance, but only PCs 1 and 2 (77.83% of the variance) were kept since PC3 showed low correlation coefficients to the variables (<|0.52|). While the number of primary and secondary branches was positively correlated to PC2, succulence was negatively correlated to PC1, and the remaining characteristics were positively correlated to PC1 (Fig. 1B). Considering the contribution of each variable to the PCs, leaf size and stem branching were important factors when separating the four clusters. The groups were composed of: A—containing exclusively *tuberculata*; B—*papillatostellulata, nitida* and *sicula*; C—*oleracea, zaffranii, trituberculata* and *rausii*; and D—*sativa*, *edulis*, and *granulatostellulata* and the cultivar (Fig. 1D,F). We tested if morphological correlated with each other (Supplementary Table S3), and positive correlations (r > 0.61, *p*-value < 0.05) revealed that large plants have large leaves and harbor more primary branches. Succulence was negatively correlated (r < − 0.59, *p*-value < 0.05) with plant robustness parameters and leaf area. Overall, taller plants harbored thinner and larger leaves, whereas smaller plants were more branched with smaller, thicker leaves (Supplementary Table S3).

When comparing the clusters formed using either the climate or morphological approaches, *tuberculata* was placed in an isolated cluster, while the following pairs clustered together using both approaches: *sicula* + *papillatostellulata*; *trituberculata* + *rausii*; and *oleracea* + *zaffranii*.

### Drought represses C_4_ and promotes CAM pathways across genotypes

**Figure 2:**
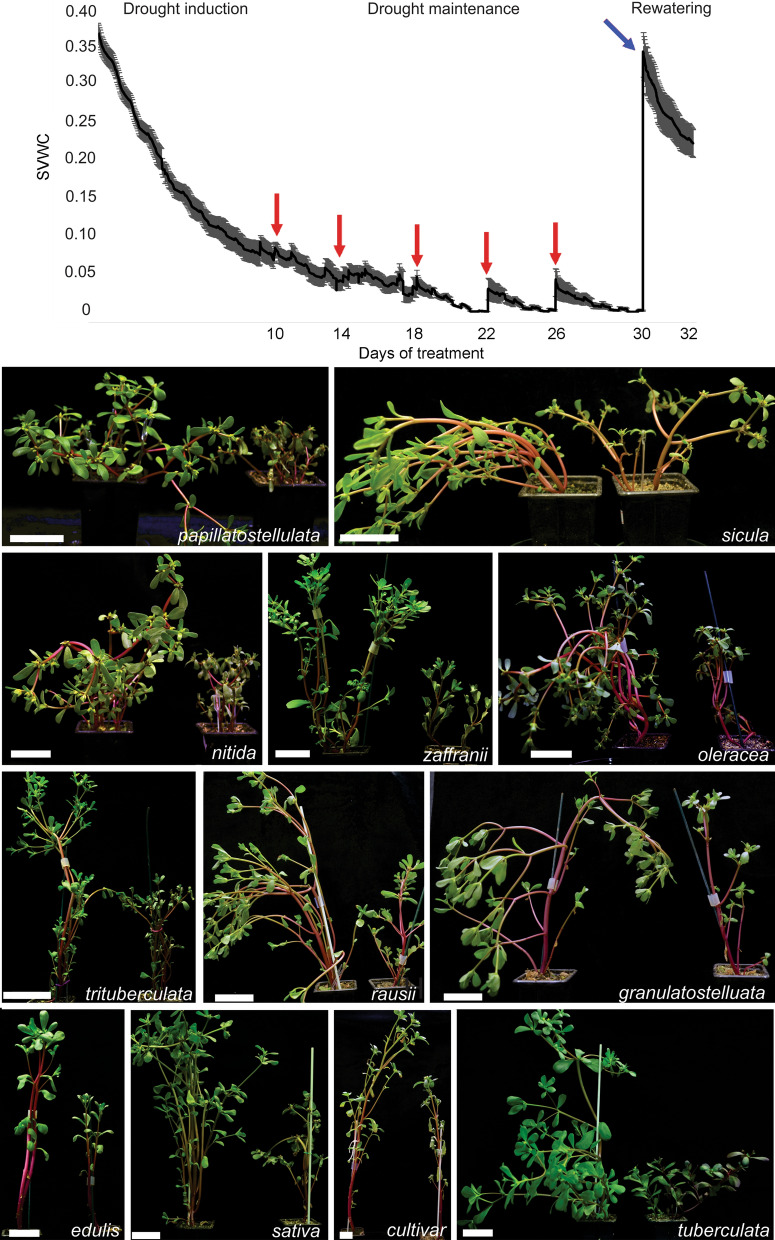
Drought treatment impacts on soil volumetric water content (SVWC) and overall plant morphology. (**A**) Changes in SVWC during drought and rewatering treatments in *P. oleracea*, with red and blue arrows indicating partial (10 ml per pot) and full watering events, respectively (see Methods for details). Data are means ± SE for monitored genotypes. (**B**) Representative images of 2-month-old plants kept under well-watered (left) and droughted (right) conditions.

After characterizing the morpho-climatic traits of our collection, the impacts of water availability on CAM photosynthesis across genotypes was investigated by comparing CAM-related traits. To this goal, one-month-old plants were kept side-by-side under well-watered or droughted conditions for 30 days, or exposed to drought for 30 days followed by two days of complete rewatering (Fig. 2A). To prevent plants from dying, the drought treatment consisted of withholding water for ten consecutive days, followed by a 20 day period in which a small water volume (10 ml) was added to the pots whenever the soil water content reached values close to zero (usually every four days). The drought treatment promoted a marked reduction in plant size in all genotypes compared to the well-watered counterparts (Fig. 2B).

**Figure 3:**
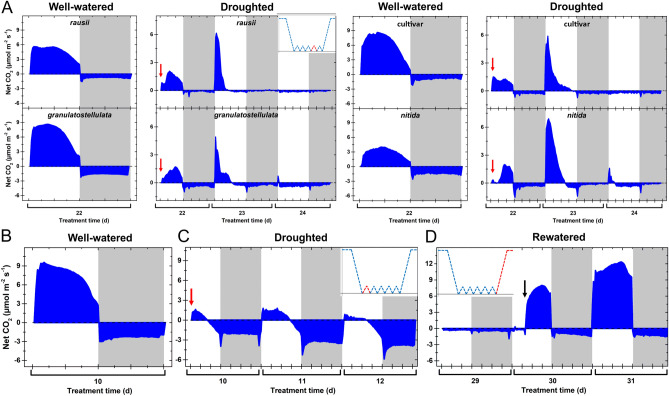
Similar drought-triggered changes in diel gas exchange are shared by *Portulaca oleracea* subspecies. (**A**) Net CO_2_ exchange of shoots of representative subspecies after 16 days of drought. (**B-D**) Net CO_2_ exchange by shoots of *granulatostellulata* in well-watered conditions (**B**), after the initial 10-day-period of water withholding (**C**), and after 20 days of drought and into rewatering (**D**). In (**A–D**), two-week-old individuals were used due to the size restriction of the cuvette. Also, data were normalized against the leaf area. Shaded areas indicate the dark period, red arrows indicate partial watering event (5 ml), and the blue arrow indicates full rewatering. Inserts schematically illustrate soil water content following water deprivation (see Methods for details), and the time points corresponding to the gas exchange measurements are highlighted in red.

We monitored continuous net CO_2_ exchange in *rausii*, *granulatostellulata*, *nitida* and the cultivar, which are representatives of each of the four morphological clusters identified in this work (Fig. 3). A multi-chamber IRGA system was used to monitor the entire shoot of 2-weeks-old plants, revealing a similar behavior for all genotypes monitored (Fig. 3). Well-watered plants displayed CO_2_ assimilation throughout the entire light period. In contrast, CO_2_ uptake was limited to a brief burst in the early morning at the start of drought treatment, whereas daytime CO_2_ uptake was undetectable at the end of the drought period. Daytime CO_2_ uptake was recovered within hours after full rewatering of the plants, indicating the activation of C_4_ photosynthesis when the water supply returned. Nocturnal net CO_2_ uptake was not observed across the genotypes analyzed.

**Figure 4:**
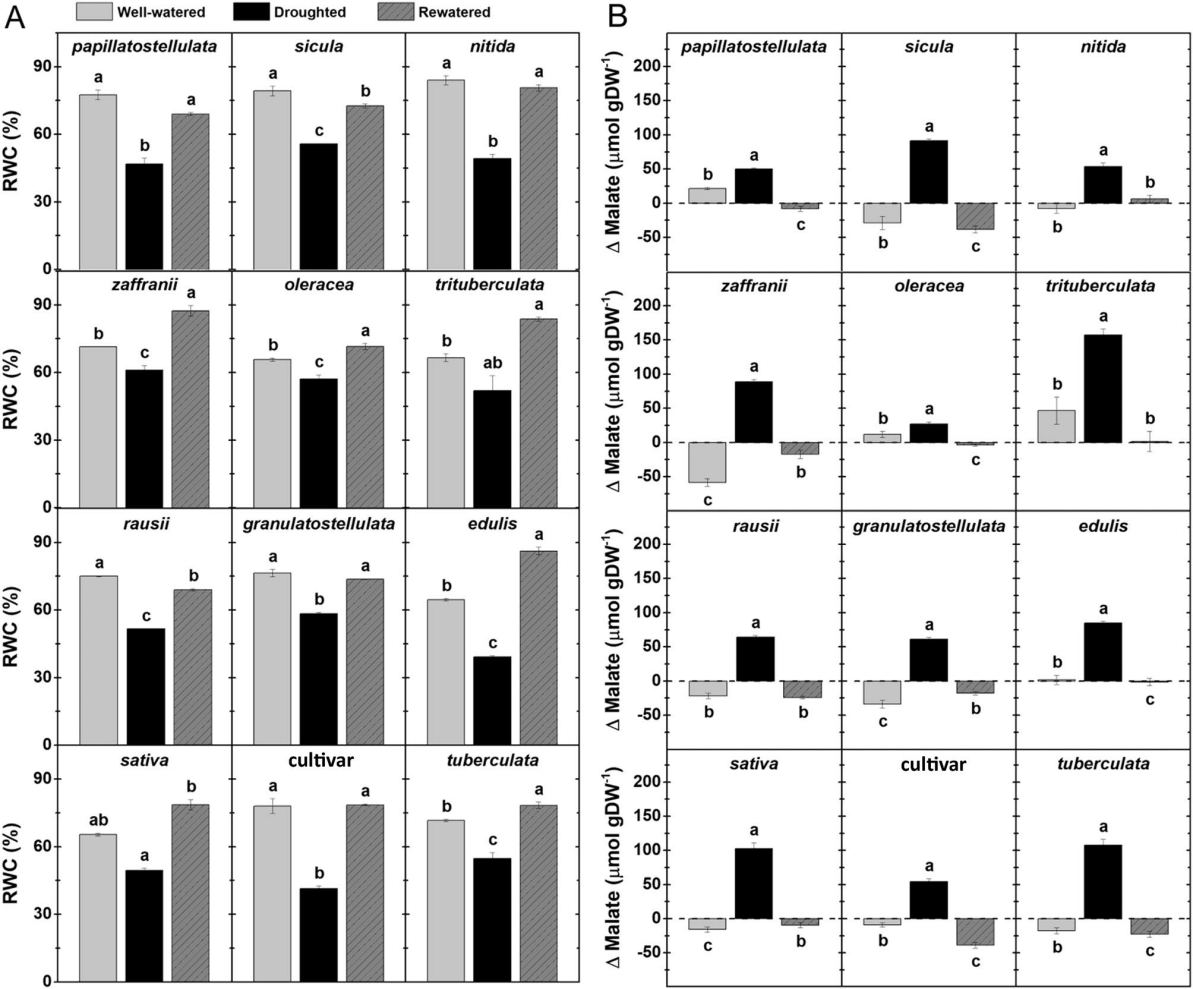
Impacts of water availability *P. oleracea* on relative water content (RWC) and nocturnal malate accumulation. (**A**) Leaf RWC of well-watered, droughted and rewatered plants. (**B**) Nocturnal malate accumulation (Δ malate) in well-watered, droughted and rewatered plants. Data are means ± SE of at least three biological replicates, and different letters indicate statistically significant differences (*p* < 0.05) among the treatments for each subspecies. In (**B**), standard error = √((standard error_well-watered_)^2^ + (standard error_droughted_)^2^.

Leaf relative water content (RWC) values were significantly lower under drought compared to well-watered conditions for all genotypes (Fig. 4A). Rewatering recovered leaf RWC to values similar, or almost similar (e.g., *sicula* and *rausii*), to those detected in well-watered plants. To investigate whether all genotypes were able to switch to CAM photosynthesis in response to drought, leaf malate levels were determined at dawn and dusk, and nocturnal malate accumulation (Δ_malate_) was calculated for each of them (Fig. 4B, Supplementary Table S4). Under well-watered conditions, *papillatostellulata*, *oleracea*, *trituberculata* and *edulis*, the latter at very low levels, displayed accumulation of malate overnight (positive Δ_malate_), whereas malate levels in the remaining genotypes decreased overnight (negative Δ_malate_) (Fig. 4B). All genotypes presented positive Δ_malate_ under drought treatment, with the lowest and highest Δ_malate_ values (26.7 and 157.2 µmol malate per g dry weight, respectively) detected in *oleracea* and *trituberculata*, respectively. Nocturnal malate accumulation was consistently reduced following rewatering in all subspecies (Fig. 4B).

**Figure 5:**
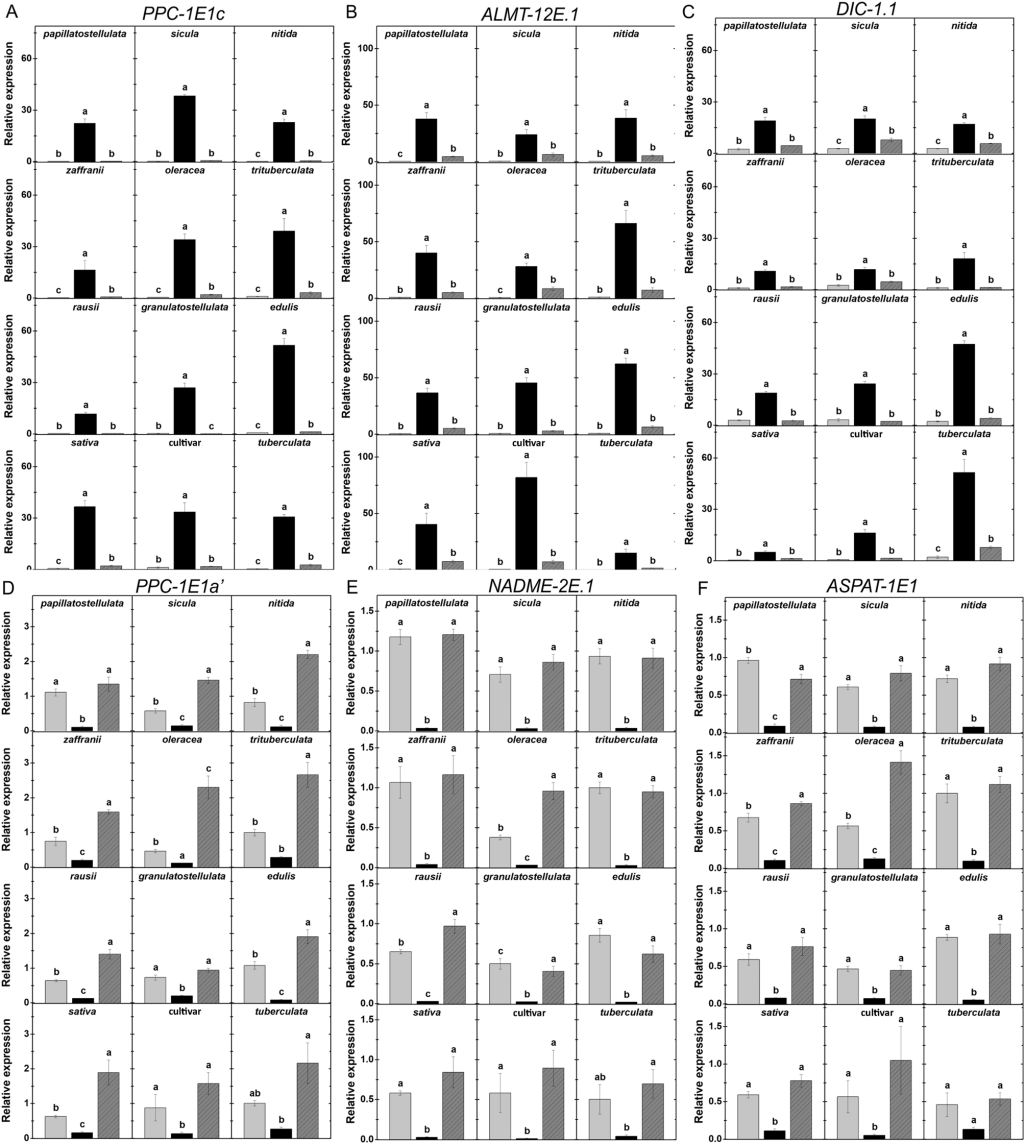
Impacts of water availability on transcript abundance of C_4_- and CAM-marker genes. (**A-C**) Relative abundance of CAM-specific transcripts: *PPC1E1c* (**A**), *ALMT-12E.1* (**B**), *DIC-1.1* (**C**). (**D–F**) Relative abundance of CAM-specific transcripts: *PPC1E1a’* (**D**), *NADME-2E.1* (**E**), *ASPAT-1E1* (**F**). Mean relative expression was normalized against well-watered *trituberculata* samples, and mRNA levels were determined in samples harvested at dawn (**A–C**) or dusk (**D–F**). Data are means ± SE of at least three biological replicates, and different letters indicate statistically significant differences (*p* < 0.05) among the treatments for each subspecies.

We then monitored the transcriptional patterns for CCM-related genes across the studied genotypes. Transcriptional profiling of CAM-related genes under drought, when compared to well-watered conditions, showed increments of up to 165, 123 and 25 times in mRNA levels of *PPC-1E1c*, *ALMT-12E.1* and *DIC-1.1*, respectively (Fig. 5A–C, Supplementary Table S4). Rewatering reduced relative transcript levels for all three CAM-marker genes significantly in most cases, reaching values as low as those detected in well-watered plants.

For C_4_-related genes such as *PPC-1E1a’*, *NADME-2E.1* and *ASPAT-1E1*, mRNA levels were between 3 and 48 times lower under drought than in well-watered plants across genotypes (Fig. 5D–F, Table S4). Rewatering resulted in *PPC-1E1a’* mRNA levels relatively higher than those detected in well-watered plants, except for *papillatostellulata*, *granulatostellulata* and the cultivar, which exhibited similar abundance in both rewatered and well-watered plants (Fig. 5F). Upon rewatering, relative transcript abundance of *NADME-2E.1* and *ASPAT-1E1* returned to levels as high as those detected in well-watered individuals (Fig. 5D–F, Supplementary Table S4).

Lastly, we assessed drought resilience across the genotypes by performing Chl *a* fluorescence imaging in source leaves of plants exposed to long-term, continuous water withholding, i.e., 20 and 35 days (Supplementary Figure S2–4). In all genotypes, PSII operating efficiency (Fq’/Fm’) was similar comparing well-watered and droughted plants, and only *trituberculata*, *rausii* and the cultivar exhibited slightly reduced Fq’/Fm’ after prolonged droughted (Supplementary Figure S2). Non-photochemical quenching (NPQ) significantly increased after drought only in *tuberculata* (Supplementary Figure S3). The most prominent reduction in maximum quantum efficiency of PSII photochemistry (Fv/Fm) was detected after 34 days of drought stress in *tuberculata*, *papillatostellulata* and the cultivar (Supplementary Figure S4).

### Comparing CCM-related physio-molecular traits across the genotypes

After characterizing C_4_–CAM photosynthesis for *P. oleracea* genotypes individually using malate quantification and transcript abundance of key C_4_–CAM-related genes, we compared the genotypes at each water availability condition separately (Supplementary Tables S4, S5). Nighttime malate accumulation was significantly different (*p*-value < 0.05) across genotypes at all three water availability conditions. However, such a pattern was not statistically supported in terms of CAM-related transcript accumulation. Although *tuberculata* clustered apart from the other genotypes at the initial morpho-climate characterization, its Δ_malate_ levels and transcript profiles did not stand out compared to the remaining genotypes, except for the lower accumulation of *ALMT-12E.1* mRNA levels under drought. Also, genotype pairs that initially clustered together (*sicula* + *papillatostellulata*; *trituberculata* + *rausii*; and *oleracea* + *zaffranii*) did not show similar trends for any of the CCM-related parameters.

Surprisingly, no significant correlations were observed between Δ_malate_ and CAM-related transcript abundance (including *PPC-1E1c* mRNA levels) under drought. However, a positive correlation (r > 0.76, *p*-value < 0.05) was observed between (1) Fv/Fm and Fq’/Fm’, (2) *PPC-1E1a'* mRNA levels and either RWC and Δ_malate_, and (3) between *NADME-2E.1* and *ASPAT-1E.1* mRNA levels (Supplementary Table S6). Also, *ALMT-12E.1* mRNA abundance was negatively correlated to *ASPAT-1E.1* transcript levels (r = − 0.74, *p*-value < 0.05, Supplementary Table S6).

## Discussion

CAM has long been described as a highly plastic adaptive syndrome, operating in different modes and magnitudes depending on the lineage^51^. CAM-related literature includes a vast array of studies on interspecific variability in the contribution of nocturnal CO_2_ uptake to net daily carbon gain (i.e., weak vs. strong CAM), the diel pattern of gas exchange, the occurrence of CAM throughout the plant life cycle or as environmental conditions change (i.e., constitutive *versus* facultative CAM), and molecular evolution of CAM-specific gene lineages^1,7,52,53^. Among CAM physiotypes, facultative CAM species, as *P. oleracea*, are recognized as particularly convenient systems for understanding the discrete changes in genetic architecture and gene expression associated with the CAM pathway^54,55^.

Different studies have already addressed the pronounced evolutionary changes forming a gradient ranging from C_3_ to obligatory CAM species^56–58^, but C_4_/CAM-performing species comprehend a new and yet little explored scenario^16,23,58,59^. In addition, studies linking CAM intensity, environmental conditions and plant morphoanatomical variations have shown that different trends may occur in different plant lineages^60^. For example, at the plant family level, some Orchidaceae show a correlation between decrescent CAM intensity and increasing altitude^61^, whereas Eulophiinae terrestrial orchids evolved higher CAM expression during the transition to drier habitats^62^. Also, tropical Oncidiinae epiphytes that express weak CAM possess thinner leaves, while strong CAM orchids have thicker leaves^63^. However, when comparing morphologically similar C_3_–CAM cycling *Talinum* species, differences in nocturnal acidity were more inconspicuous, although correlated with low humidity coefficients (r = − 0.55) from each species place of sampling^64^. Although comparing leaf thickness and cell size from C_3_ and obligate CAM *Yucca* species showed a positive correlation to nocturnal gas exchange and higher leaf acidification^65^, the comparison of 24 genotypes of C_3_–CAM *Yucca gloriosa* showed no correlation between leaf anatomy and CAM intensity^66^. This highlights that among intermediate phenotypes, the evolutionary trends may be more challenging to identify. Therefore, each plant lineage may show specific trends for the group, not necessarily matching the typical CAM traits as traditionally described^67^.

In this context, the *P. oleracea* complex represents a valuable system both for exploring the intraspecific variability of CAM and for providing additional biochemical and genetic information about the rare co-occurrence of C_4_ and CAM pathways. Traditionally, *P. oleracea* has been considered an aggregate of subspecies or microspecies^43,46,47^, also sometimes referred to as different species^68–70^. To our knowledge, taxonomic reports list 19 subspecies/microspecies distinguished according to their seed size and coat ornaments^46^. On the other hand, *P. oleracea* is sometimes considered a polymorphic species, and due to its cosmopolitan distribution and high adaptability, it is somewhat expected to present high variability in morphological traits among populations, even forming a continuum^33,37,38,71^. Such plasticity might also affect seed attributes, making seed morphology and size alone inconclusive to differentiate subspecies, especially considering that hybrid subspecies have already been reported in mixed populations^33,37^. A comprehensive phylogeny including as many accessions of *P. oleracea* as possible will be needed to solve the species paraphyletic scenario, but its cosmopolitan distribution may prove to be a challenge to this goal. In the present study, we selected genotypes sampled from independent populations and identified as different subspecies, and the clustering methods applied here confirmed previously described trends^38^, where weedy (small, prostrate and branched) phenotypes were clustered separately from more robust and erect phenotypes, e.g. commercial cultivars.

Regarding the intraspecific metabolic plasticity in *P. oleracea*, CAM was found to be expressed in a completely reversible way in all genotypes analyzed in the present study. Our findings indicate that, although there are significant intraspecific differences in drought-induced Δ_malate_, these are not directly correlated with the transcript abundances of CAM-specific genes (*PPC-1E1c, ALMT-12E.1* and *DIC-1.1*). Also, there was no correlation between the transcript levels of these CAM-related genes that would suggest a causal relation. Therefore, the widespread occurrence of low-level CAM expression across the *P. oleracea* genotypes seems to be achieved without a strict balance between the expression levels of key CAM-related genes and the intensity of nighttime acidification, which further reflects the complexity behind the CAM syndrome^7,55^.

Over the last years, molecular and bioinformatics tools have been progressively applied to characterize the large gradient of expression found in CAM plants^72–77^. Thus, understanding the molecular processes behind CAM photosynthesis has gained further interest as a source of information for bioengineering endeavors seeking to improve crop resistance to extreme drought conditions^49,78,79^. However, a comparative study across different genotypes using CCM transcript abundances of key genes in C_4_–CAM species was missing, despite its potential to provide relevant information for future attempts of engineering CAM into C_4_^26,49^_._ Here we show that, at least at the transcriptional level, major components of C_4_ and CAM photosynthesis were regulated in opposite directions by water availability across all *P. oleracea* genotypes analyzed. All three CAM-marker genes (*PPC-1E1c, ALMT-12E.1* and *DIC-1.1*^27^), previously identified exclusively in one genotype (here referred to as cultivar), were expressed at significantly high levels under drought in all genotypes analyzed. This indicates that, in overall terms, the transcriptional control of key components of CAM machinery by water availability is conserved within the *P. oleracea* complex. Similarly, the C_4_-related genes *PPC-1E1a’*, *NADME-2E.1* and *ASPAT-1E1* were also clearly down- and up-regulated across subspecies by drought and rewatering, respectively. Still, individual puzzle pieces belonging to different CCMs, such as the significant negative correlation between *ALMT-12E.1* (CAM) and *ASPAT-1E.1* (C_4_) mRNA levels under drought, indicates that there is still much room for investigating the intricate connection and concomitant modulation between C_4_ down- and CAM up-regulation.

Weak, inducible CAM has been reported for different *Portulaca* species, with the drought-promoted increase in nocturnal acidification either associated with a small nocturnal CO_2_ uptake (e.g. *P. cyclophylla* and *P. cryptopela*) or as a result of CAM-cycling (e.g. *P. digyna*)^15,16^. The diel CO_2_ uptake patterns observed in the present study support the occurrence of CAM-cycling for all four *P. oleracea* analyzed. Therefore, it is safe to assume that the malate formed overnight in droughted *P. oleracea* genotypes is derived from recycling nocturnal respiratory CO_2_^54^ . The drought-induced Δ_malate_ values reported here for *P. oleracea* are comparable to previous works detecting malate in the same species^21^, and close to the range of acidity detected for other droughted *Portulaca* species (e.g. *P. digyna* and *P. cyclophylla* showed 45.8 and 8.5 µmol H^+^ gFW^−1^, respectively^14^). Overall, when considering a combination of morpho-groups and Δ_malate_ values under drought stress, a small array of combinations was formed, with robust (*sativa*—102.6 µmol gDW^−1^ and *granulatostellulata*—60.94 µmol gDW^−1^) and weedier (*sicula*—91.15 µmol gDW^−1^ and *papillatostellulata*—50.13 µmol gDW^−1^) phenotypes with representatives showing contrasting Δ_malate_ values.

Facultative CAM may provide adaptive advantages other than carbon gain, even for weak cyclers^80^. In this context, the drought-resilient phenotypes of *P. oleracea* reported here, confirmed by the maintenance of photosystem operation throughout prolonged drought (Supplementary Figure S2–S4), and its full and rapid recovery of C_4_ upon rewatering (Fig. 3D), may be supported by the persistence of daytime CO_2_ assimilation behind closed stomata from the decarboxylation of malate accumulated overnight^80,81^. Also, without undermining the contribution of other traits (e.g., abundant seed production, resistance to abiotic stresses)^82^, the growth rates offered by C_4_ photosynthesis when water is available, combined with drought resilience facilitated by CAM expression under drought, can contribute to the weediness of *P. oleracea*. Other morpho-physiological traits as differences in water-capture strategies, cuticle thickness, epicuticular wax, stomatal density, stomatal responsiveness and root architecture, which remains to be determined for the subspecies, may be behind the remarkable drought resilience observed across the *P. oleracea* complex. Moreover, the high antioxidant capacity typically found in *P. oleracea* leaves^24,25^, may also be particularly important to maintain photosystem operation and avoid oxidative damage during severe drought spans.

In conclusion, drought was shown to simultaneously downregulate C_4_ and promote CAM in all *P. oleracea* genotypes. The mode of CAM expression (i.e., weak, facultative CAM, CAM-cycling), and the C_4_/CAM-marker gene expression profiles were conserved across the genotypes, further emphasizing the occurrence of inducible CAM as a common trait shared by all members of the *Portulaca* genus. As facultative C_4_/CAM photosynthesis is found across *P. oleracea* complex, future studies on the tissue organization and molecular regulatory requirements for the occurrence of concomitant C_4_/CAM photosynthesis could be performed in genotypes with significant morphological differences. Such studies may provide critical insights for future attempts aiming at incorporating C_4_/CAM into crop species. Moreover, from the perspective of photosynthetic plasticity, virtually any of the 12 genotypes of *P. oleracea* analyzed here could serve as model systems for further studies on C_4_–CAM transition. Therefore, other aspects, including plant morphology, genome size, ploidy level, and amenability to genetic transformation, could be taken into consideration before the selection of a particular *P. oleracea* genotype to become a genetic model for C_4_–CAM research.

## Materials and methods

### Studied *Portulaca oleracea* subspecies

To investigate the potential differences in CAM plasticity within the *P. oleracea* complex, a collection of twelve purslane accessions from different geographical locations was assembled (Table 1). Although seed size is not trustworthy for subspecies identification, seed ornamentation is so far considered the best-described structural attribute for subdividing of the *P. oleracea* complex^46^, allowing cross-referencing with previous studies in this species. Therefore, at least 15 seeds from each genotype were critical point-dried (Balzers CPD 030), coated with gold (Balzers SCD 050 Sputter Coater), and examined in a scanning electron microscope (Sigma VP—Zeiss, Oberkochen, Germany) to confirm the purity of the lots. The status for each subspecies was checked at The Plant List^83^ (Table 1). The genotype here referred to as “cultivar” was previously used in transcriptome studies^27^ and was included in this study as a reference for physiological and molecular data. Seeds from each accession were grown under well-watered conditions for at least three generations before the experiments.

### Morphometric analysis

Morphometric measurements were performed in at least 25 individuals of each subspecies grown for 8–10 weeks under well-watered conditions. The following parameters were selected based on existing literature^38^ and analyzed: plant height (cm), fresh plant weight (g), number of primary and secondary branches, diameter of stem base and apex (cm, measured at approximately 1 cm from plant edges and used to calculate stem base/apex ratio), total and mean leaf area (cm^2^), leaf length and width (used to calculate leaf format ratio), and succulence (saturated water content^84^).

### Plant material and growth conditions for drought experiments

Plants were grown in 300-mL pots containing a 1:1 mixture of commercial substrate (Plantmax HT, Eucatex, São Paulo, Brazil) and expanded vermiculite supplemented with 1 g L^−1^ NPK 10:10:10, 4 g L^−1^ of dolomite limestone (MgCO_3_ + CaCO_3_) and 2 g L^−1^ thermophosphate (Yoorin Master, Brazil). Plants were kept in a growth chamber at approximately 600 µmol m^2^ s^−1^ incident to the top of the chamber, 12 h photoperiod, air temperature of around 27 °C day/22 °C night and air humidity of approximately 60% day/80% night. All plants were watered daily to field capacity until the start of the treatments.

For assessing CAM plasticity, one-month-old plants were separated into three experimental groups subjected to different watering regimes: (1) well-watered, (2) droughted, and (3) rewatered. Well-watered plants were continuously irrigated to field capacity throughout the experiment. Water was withheld from droughted and rewatered groups for 10 days, and subsequently, 10 mL water was added per pot whenever soil humidity reached values close to zero, usually every 4 days, for 20 consecutive days. Plants of the rewatered group were irrigated to field capacity for the next 4 days. From the start of the drought treatment, soil volumetric water content (SVWC) was continuously monitored using Decagon soil moisture meter EC-5 (Fig. 2). At the end of each treatment, samples were harvested 1 h after the onset of illumination (dawn samples) and 1 h before the end of the light period (dusk samples). For all analyses, four biological replicates, each replicate composed of all fully-expanded and non-senescent leaves of at least three plants, were harvested at each sampling time. Samples were frozen, powdered and stored at − 80 °C until use. Plants from all genotypes were grown side-by-side, and downstream analyses were performed with samples for all genotypes at the same time to avoid introducing unnecessary variation.

### Relative water content (RWC)

Fresh weight (FW) was determined in 10 leaf discs (~ 0.8 cm diameter) immediately after harvesting. Subsequently, the leaf discs were fully-hydrated by incubation in deionized water for 24 h, followed by measuring the turgid weight (TW). Finally, the samples were dried to a constant weight at 65 °C and allowed to cool down before determining the dry weight (DW). RWC was calculated using the formula [(FW − DW)/(TW − FW) × 100]^85^.

### Organic acids quantification

For organic acid profiling, approximately 200 mg FW of frozen leaf samples were extracted in 1 ml of 80% (v/v) ethanol for 15 min at 80 °C, and the supernatants were recovered by centrifugation (5,000 g, 15 min). Pellets were re-extracted three times, and all supernatants were combined and reduced to dryness under vacuum. Aliquots of 1 mL of the supernatant were dried under vacuum and resuspended in 300 µL ultrapure water. Chromatography was carried out on an Agilent Technologies series 1,200 coupled with a diode array detector (DAD) on a reverse-phase column (SupelcoGel C610H-6% Cross Linked HPLC Column 300 mm × 7.8 mm, 9 µm) and with a guard column (SupelcoGel H Guard Column 50 mm × 4.6 mm, 9 µm) at 30 °C, using 0.1% (v/v) phosphoric acid as mobile phase running isocratically at 0.5 mL min^−1^. Eluted compounds were detected at 210 nm and quantified through external calibration. Endogenous metabolite concentrations were obtained by comparing the peak areas of the chromatograms against commercial standards.

### Continuous gas exchange

Gas exchange was monitored using a 12-channel, custom-built IRGA system (PP Systems), as described previously^86^. The twelve cuvettes were housed in a growth chamber at approximately 600 μmol m^−2^ s^−1^ incident to the top of the chamber, 12 h photoperiod, air temperature of 28 °C day/18 °C night and air humidity of 60% day/80% night. Gas exchange was monitored using an infra-red gas exchange system based on a CIRAS-DC analyzer (PP Systems, USA), and calculated using SC-DC software (PP Systems, USA). The contribution of soil respiration and soil moisture to the environment in the cuvette was minimized by wrapping multiple-layers of parafilm around the rim of the pot, with a little hole for the stem. CO_2_ exchange rates were based on leaf area, measured using ImageJ 1.50i (NIH, Bethesda, MD, USA).

### Chlorophyll a fluorescence imaging

The maximum quantum efficiency of PSII photochemistry (Fv/Fm), PSII operating efficiency (Fq’/Fm’) and non-photochemical quenching (NPQ) were determined through a non-modulated imaging fluorometer (CF Imager, Technologica, UK), as described by Baker (2008). All measurements were taken between 2 and 5 h after the start of the light period, and values of minimal (Fo) and maximal (Fm) fluorescence were obtained from dark-adapted leaves for 30 min before receiving a saturating light pulse (~ 6,000 µmol photons m^-2^ s^-1^ for 1 s). Measurements were performed in at least two leaves from three different plants for each treatment.

### RNA isolation and quantitative RT-PCR (qPCR) analysis

Total RNA was extracted from approx. 80 mg of frozen leaves using the ReliaPrep RNA Tissue Miniprep System (Promega) for fibrous tissues, with careful homogenization steps. RNA samples were quantified using a microvolume spectrophotometer (NanoDrop ND-1000, Thermo Scientific, USA). Purity was assessed by keeping ratios in between the following intervals: 1.8 < A_260/280_ < 2.3; 1.6 < A_260/230_ < 2.3. The extracted RNA was treated with DNase (DNase I Amplification Grade, Thermo Fisher Scientific) for 10 min at room temperature. Complementary DNA (cDNA) synthesis was synthesized using SuperScript IV Reverse Transcriptase kit (Thermo Fisher Scientific) and qPCR reactions were performed in a StepOnePlus Real-Time PCR System (Applied Biosystems), using 10 μl mix reaction composed of 5 μl Power SYBR green 2X (Thermo Fisher Scientific), 2 μl cDNA sample and 300 nM of forward and 300 nM of reverse primers. The amplification program consisted of 10 min initial step at 95 °C, followed by 40 cycles with 15 s 95 °C, 30 s 60 °C and 30 s 72 °C. In all cases, the melting curve was analyzed to detect unspecific amplification and primer dimerization. The relative transcript abundance was calculated by applying the 2^−ΔΔCT^ method (Livak and Schmittgen 2001). All primer sequences used are listed in Supplementary Table S7.

### Climate space data

Using the coordinates from their places of sampling (Table 1), climate information (19 variables) for all 11 subspecies was retrieved from the WorldClim database (https://www.worldclimorg/bioclim)^50^ at a spatial scale of 10 min of a degree, using the raster package^87^ in R 3.6.1^88^.

### Statistical analysis

All statistical analyses were performed using R (version 3.6.1^88^) via RStudio (version 1.2.1335). The data were checked for normality using the Shapiro–Wilk test, and for homogeneity of variances using the Levene test. When appropriate, two means were compared using: two-sample t-test (normal, homoscedastic), Welch’s t-test (normal, heteroscedastic), Mann–Whitney test (non-normal, homoscedastic) or transformed by square root or log and fitted into any of the other descriptions (non-normal, heteroscedastic). For comparison between three or more means, we used: ANOVA and post-hoc Tukey test (normal, homoscedastic), Welch ANOVA + Games-Howell post-hoc test (normal, heteroscedastic); Kruskal–Wallis + Mann–Whitney U-paired post-hoc test without adjusted *p*-value (non-normal, homoscedastic) or transformed by square root or log and fitted into any of the other descriptions (non-normal, heteroscedastic).

Morphometric and climate data were normalized using z-scale and analyzed by principal component analysis (PCA) using the pre-installed R function prcomp() (visualized using factoextra package^89^). Hierarchical clustering analysis was done using the pvclust package (version 2.0-0^90^) with Euclidean distance and the Ward’s method criterion. For each dendrogram, AU (Approximately Unbiased) and BP (Bootstrap Probability) values were computed using 1,000 bootstraps—see pvclust manual (Available at: https://cran.r-project.org/web/packages/pvclust/pvclust.pdf). The AU value is an unbiase p-value, more accurate than BP.

## Supplementary information

Supplementary tables.

Supplementary figures.
