# Supplementary material for: Exploring C4–CAM plasticity within the Portulaca oleracea complex
Source: Sci Rep. 2020 Aug 28;10:14237. doi: 10.1038/s41598-020-71012-y (PMC7455729; doi:10.1038/s41598-020-71012-y)
Supplement: Supplementary file 2 — Supplementary figures. [file 41598_2020_71012_MOESM2_ESM.docx]

**Supplementary Information**

Title: Exploring C_4_-CAM plasticity within the *Portulaca oleracea* complex

Authors: Renata Callegari Ferrari, Bruna Coelho Cruz, Vinícius Daguano Gastaldi, Thalyson Storl, Elisa Callegari Ferrari, Susanna F. Boxall, James Hartwell, Luciano Freschi

List of files included:

| Fig. S1. Seed coat ornamentation of *P. oleracea* subspecies studied under scanning electron microscopy (SEM). |
| --- |
| Fig. S2. Impacts of drought on PSII operating efficiency (Fq’/Fm’) of *P. oleracea* subspecies. |
| Fig. S3. Impacts of drought on non-photochemical quenching (NPQ) in *P. oleracea* subspecies. |
| Fig. S4. Impacts of drought on maximum quantum efficiency of PSII photochemistry (Fv/Fm) in *P. oleracea* subspecies. |
| Table S1. GPS coordinates and climatic variables for the places of origin of eleven *P. oleracea* subspecies. (Separate Excel File) |
| Table S2. Morphometric attributes of *P. oleracea* genotypes. (Separate Excel File) |
| Table S3. Correlation coefficient (r) matrix for morphometric parameters compared across *P. oleracea* genotypes. (Separate Excel File) |
| Table S4. CCM-related parameters for 11 subspecies and one cultivar of *P. oleracea*. (Separate Excel File) |
| Table S5. P-values for CCM-related parameters for 11 subspecies and one cultivar of *P. oleracea*. (Separate Excel File) |
| Table S6. Correlation coefficient (r) matrix for C_4_-CAM-related parameters compared across *P. oleracea* genotypes. (Separate Excel File) |
| Table S7. Primer sequences used for RT-qPCR. (Separate Excel File) |


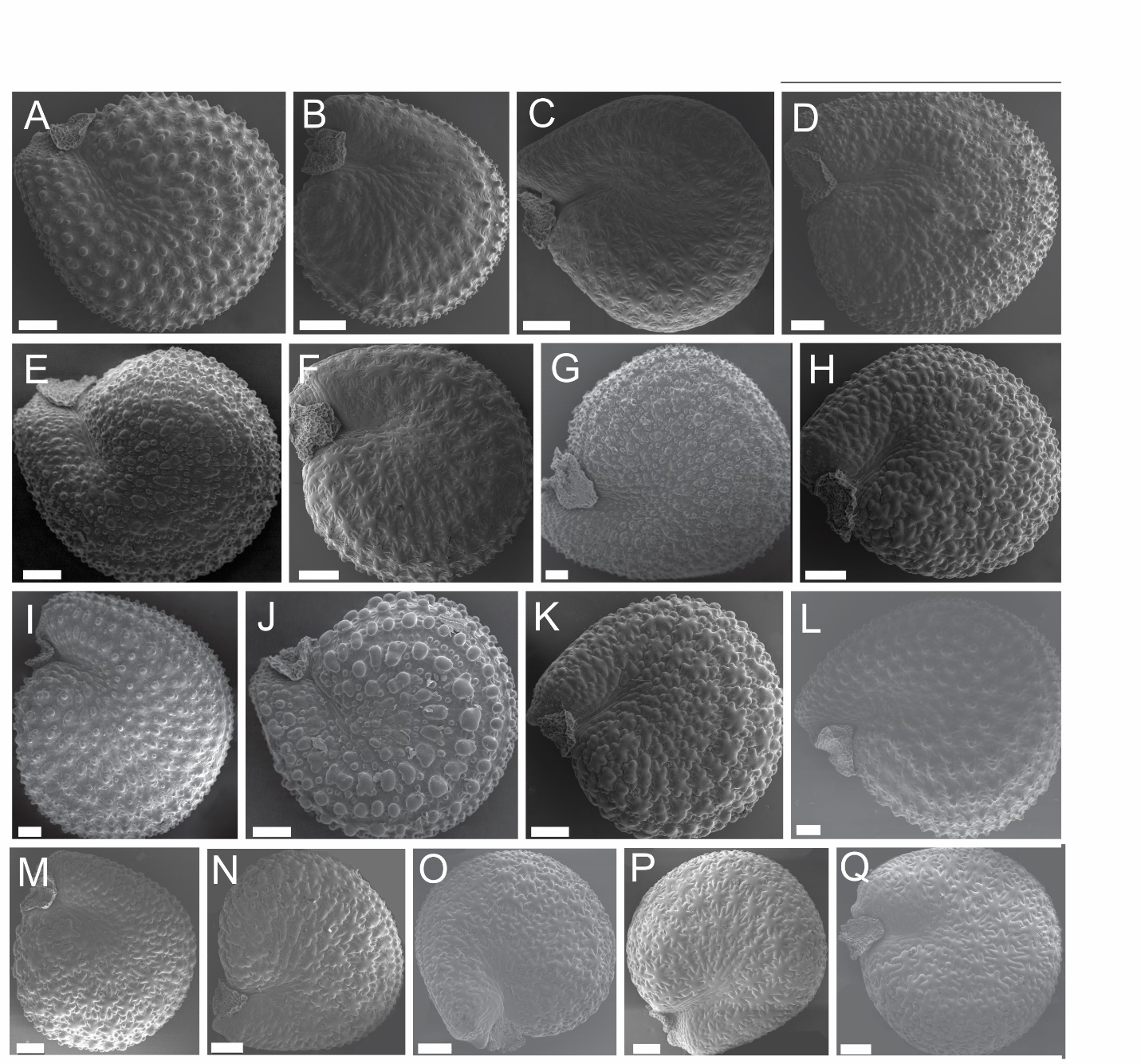


Figure S1. Seed coat ornamentation of *P. oleracea* subspecies studied under scanning electron microscopy (SEM). At least fifteen seeds of each genotype were randomly analyzed. (A) subsp. *trituberculata*. (B) subsp. *sicula*. (C) subsp. *oleracea*. (D) subsp. *rausii*. (E) subsp. *zaffranii*. (F) subsp. *nitida*. (G) subsp. *edulis.* (H) subsp. *papillatostellulata*. (I) subsp. *sativa*. (J) subsp. *tuberculata*. (K) subsp. *granulatostellulata*. (L) commercial *cultivar*. (M-N) Brazilian accessions identified as subsp. g*ranulatostellulata*. (O-Q) Brazilian accessions identified as subsp. *nitida .* Scale bars = 100 μm.


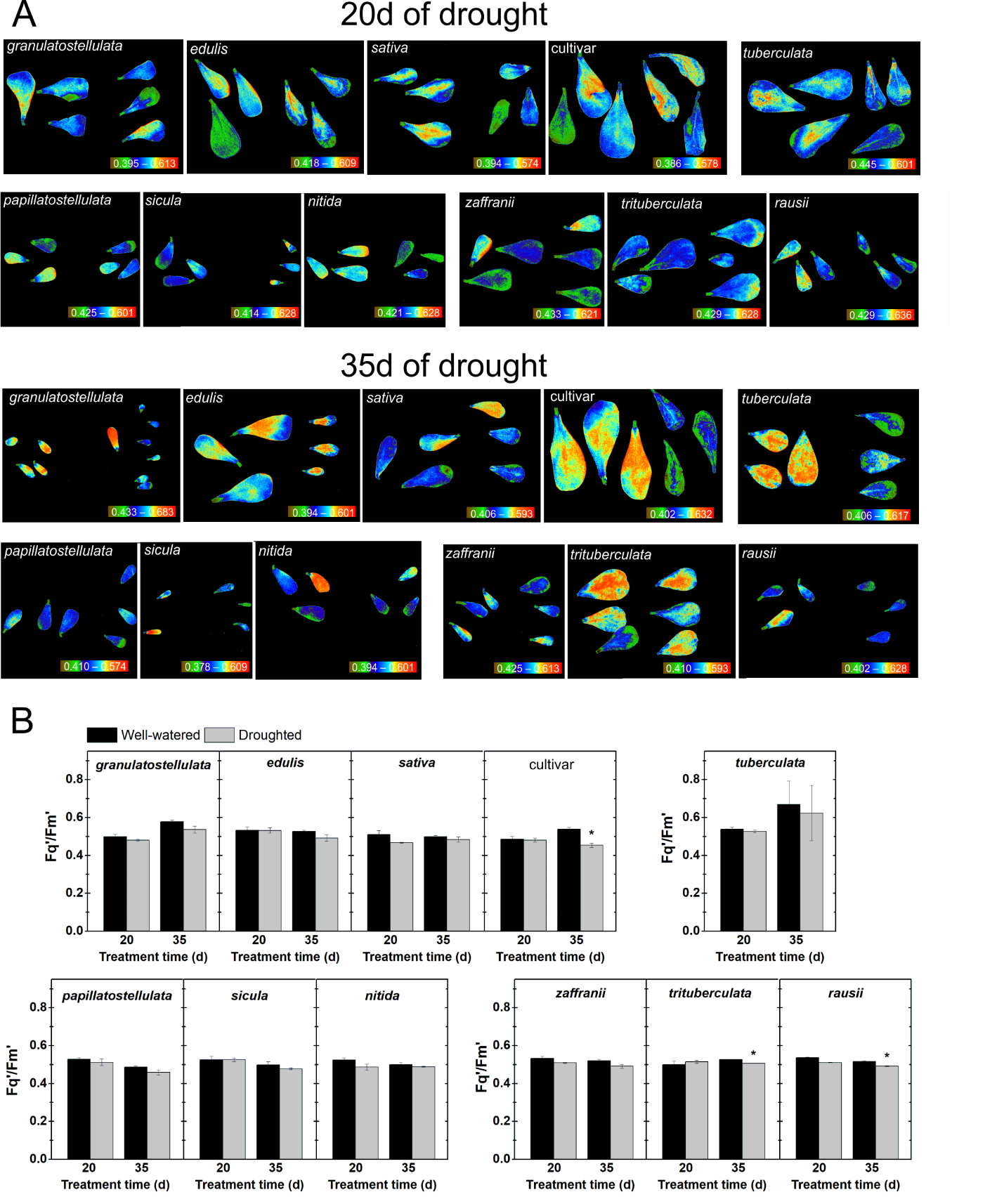


**Figure S2**. Impacts of drought on PSII operating efficiency (Fq’/Fm’) of *P. oleracea* subspecies. One-month-old plants were either kept under well-watered conditions (left) or prolonged, continuous drought (right) for 20 and 34 days. (A) Representative images. (B) Mean Fq’/Fm’ values from at least two leaves from three plants. Bars indicate SE and asterisks indicate statistical differences (p < 0.05) between treatments for each subspecies.


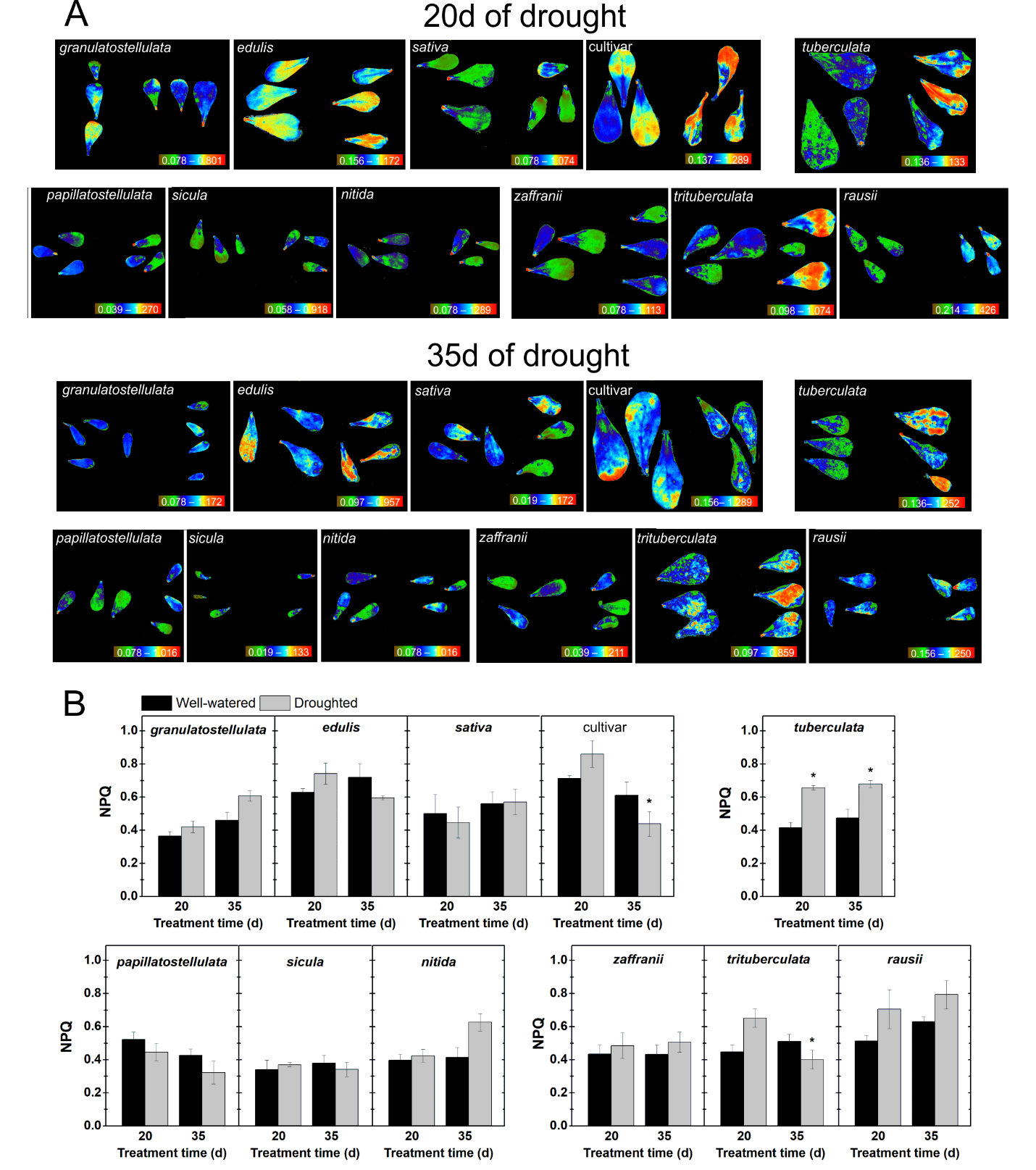


**Figure S3.** Impacts of drought on non-photochemical quenching (NPQ) in *P. oleracea* subspecies. One-month-old plants were either kept under well-watered conditions (left) or prolonged, continuous drought (right) for 20 and 34 days. (A) Representative images. (B) Mean NPQ values from at least two leaves from three plants. Bars indicate SE and asterisks indicate statistical differences (p < 0.05) between treatments for each subspecies.


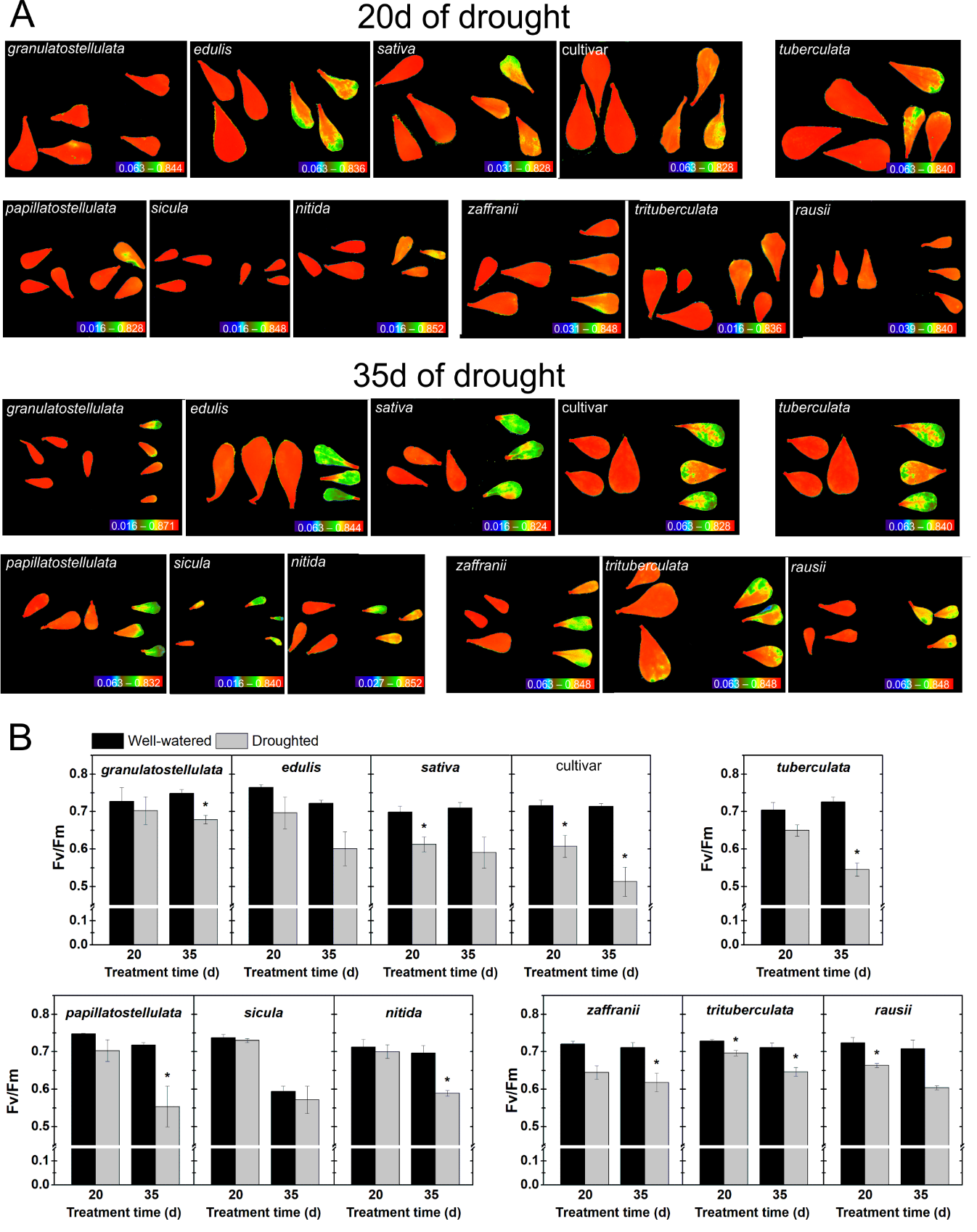


**Figure S4**. Impacts of drought on maximum quantum efficiency of PSII photochemistry (Fv/Fm) in *P. oleracea* subspecies. One-month-old plants were either kept under well-watered conditions (left) or prolonged, continuous drought (right) for 20 and 34 days. (A) Representative images. (B) Mean NPQ values from at least two leaves from three plants. Bars indicate SE and asterisks indicate statistical differences (p < 0.05) between treatments for each subspecies.
